# IL-1 Signal Inhibition In Alcoholic Hepatitis (ISAIAH): a study protocol for a multicentre, randomised, placebo-controlled trial to explore the potential benefits of canakinumab in the treatment of alcoholic hepatitis

**DOI:** 10.1186/s13063-021-05719-2

**Published:** 2021-11-11

**Authors:** N. Vergis, V. Patel, K. Bogdanowicz, J. Czyzewska-Khan, F. Fiorentino, E. Day, M. Cross, N. Foster, E. Lord, R. Goldin, E. Forrest, M. Thursz

**Affiliations:** 1 Department of Metabolism, Digestion and Reproduction, Imperial College London, London, UK; 2 Institute of Liver Studies, King’s College Hospital NHS Foundation Trust, London, UK; 3 Institute of Hepatology London, Foundation for Liver Research, London, UK; 4 School of Immunology and Microbial Sciences, Faculty of Life Sciences and Medicine, King’s College London, London, UK; 5 Imperial Clinical Trials Unit, Department of Surgery and Cancer, Imperial College, London, UK; 6 Glasgow Royal Infirmary and University of Glasgow, Glasgow, UK

**Keywords:** Alcoholic hepatitis, Interleukin 1β, Canakinumab

## Abstract

**Background:**

Alcohol consumption causes a spectrum of liver abnormalities and leads to over 3 million deaths per year. Alcoholic hepatitis (AH) is a florid presentation of alcoholic liver disease characterized by liver failure in the context of recent and heavy alcohol consumption. The aim of this study is to explore the potential benefits of the IL-1β antibody, canakinumab, in the treatment of AH.

**Methods:**

This is a multicentre, double-blind, randomised placebo-controlled trial. Participants will be diagnosed with AH using clinical criteria. Liver biopsy will then confirm that all histological features of AH are present. Up to 58 participants will be recruited into two groups from 15 centres in the UK. Patients will receive an infusion of Canakinumab or matched placebo by random 1:1 allocation. The primary outcome is the difference between groups in the proportion of patients demonstrating histological improvement and will compare histological appearances at baseline with appearances at 28 days to assign a category of “improved” or “not improved”. Patients with evidence of ongoing disease activity will receive a second infusion of canakinumab or placebo. Participants will be followed up for 90 days. Secondary outcomes include mortality and change in MELD score at 90 days.

**Discussion:**

This phase II study will explore the benefits of the IL-1β antibody, canakinumab, in the treatment of AH to provide proof of concept that inhibition of IL-1β signalling may improve histology and survival for patients with AH.

**Trial registration:**

EudraCT 2017-003724-79. Prospectively registered on 13 April 2018.

## Administrative information

Note: the numbers in curly brackets in this protocol refer to SPIRIT checklist item numbers. The order of the items has been modified to group similar items (see http://www.equator-network.org/reporting-guidelines/spirit-2013-statement-defining-standard-protocol-items-for-clinical-trials/).

**Table:** 

Title {1}	IL-1 Signal Inhibition in Alcoholic Hepatitis (ISAIAH)
Trial registration {2a and 2b}.	Prospectively registered with EudraCT 2017-003724-79.
Protocol version {3}	5.0 3^rd^ September 2020 (latest version)
Funding {4}	Novartis Pharmaceuticals UK Ltd provided funding and drug product support with clinical trial supplies of canakinumab and matching placebo. Novartis did not have any involvement in study design or study conduct.
Author details {5a}	Vergis N^1^, Patel V^2,3,4^, Bogdanowicz K^5^, Czyzewska-Khan J^5^, Fiorentino F^5^, Day E^5^, Cross M^5^, Foster N, Lord E^1^, Goldin R^1^, Forrest E^6^, Thursz M^1^ ^1^Department of Metabolism, Digestion and Reproduction, Imperial College London ^2^Institute of Liver Studies, King’s College Hospital NHS Foundation Trust, ^3^Institute of Hepatology London, Foundation for Liver Research ^4^School of Immunology and Microbial Sciences, Faculty of Life Sciences and Medicine, King’s College London, ^5^Imperial Clinical Trials Unit, Department of Surgery and Cancer, Imperial College, London ^6^Glasgow Royal Infirmary and University of Glasgow
Name and contact information for the trial sponsor {5b}	Ruth Nicholson Jrco.ctimp.team@ic.ac.uk Imperial College, London Room 221, Medical School Building, St Mary’s Campus, Norfolk Place, London W2 1PG
Role of sponsor {5c}	Authors of this manuscript are employees of Imperial College, the study sponsor, and have played a role in study design; collection, management, analysis, and interpretation of data; writing of the report; and the decision to submit the report for publication. The sponsor will have ultimate authority over these activities.

## Introduction

### Background and rationale {6a}

Alcohol consumption causes a spectrum of liver abnormalities and leads to over 3 million deaths per year [1]. Alcoholic hepatitis (AH) is a florid presentation of alcoholic liver disease characterized by liver failure (jaundice and coagulopathy) in the context of recent and heavy alcohol consumption [2]. The condition carries a high fatality risk; patients with severe AH have a 30% mortality rate at 90 days after presentation. Currently, there are no effective treatments for AH.

The most closely studied therapy is the corticosteroid prednisolone. In the largest study in this population to date of 1103 patients in 2015, prednisolone use was not associated with survival benefit compared to placebo at any timepoint [3]. In recent Cochrane meta-analyses, prednisolone was not associated with a survival benefit at any timepoint [4, 5]. In other meta-analyses, a benefit was reported at 28 days but beyond this timepoint a higher incidence of infection resulted in no survival advantage at 90 days compared to placebo treatment [6]. In no individual study or meta-analysis is there evidence of benefit beyond 1 month [7, 8]. In view of these data, clinical guidelines recommend that prednisolone may be considered for use for patients with AH [9] but do not mandate its use. Predicting which patients are at risk of prednisolone-associated infection is uncertain [10]. Moreover, combining prednisolone with anti-cytokine therapy has led to high rates of life-threatening infections in previous studies [11].

AH is a clinical syndrome associated with steatohepatitis on liver histology. Severity of the disease is graded according to Maddrey’s discriminant function (mDF) (based on bilirubin and prothrombin time), Glasgow alcoholic hepatitis score (based on age, white blood cell count, urea, prothrombin time and bilirubin) or Model for End-Stage Liver Disease (MELD) score (based on creatinine, bilirubin and INR) [2]. Alcoholic hepatitis is classified as severe when the mDF is ≥ 32. In the STOPAH trial, severe AH patients with MELD ≤ 25 and MELD ≤ 27 was less susceptible to infection even when treated with prednisolone, with 90-day mortality rates of 18% and 22%, respectively [12].

#### Rationale for IL-1 inhibition in AH

IL-1 levels are significantly increased in both serum and liver in patients with alcoholic hepatitis as well as in animal models of the disease. Patients with AH have serum levels almost 10 times higher than those found in healthy controls [13]. IL-1 is thought to be responsible for many of the clinical and metabolic characteristics of alcoholic hepatitis including fever, neutrophilia, monocyte activation, anorexia and muscle catabolism, metabolic disturbances, fibrogenesis stellate cell activation and consequently portal hypertension [14]. In animal models, inhibition of IL-1 signalling considerably attenuates disease severity [15, 16].

IL-1 inhibition using canakinumab is currently licensed therapy for cryopyrin-associated periodic syndromes, Tumour necrosis factor Receptor Associated Periodic Syndrome (TRAPS), hyperimmunoglobuminaemia D syndrome (HIDS), systemic juvenile idiopathic arthritis, familial Mediterranean fever (FMF) and gouty arthritis. Canakinumab has demonstrated a favourable risk-benefit profile in patients with these diseases. Previous studies with anti-TNFα monoclonal antibodies have not shown benefit due to the increased risk of infection and possibly due to the removal of a regenerative signal provided by TNFα [11, 17].

### Objectives {7}

To explore the benefits of the IL-1β antibody, canakinumab, in the treatment of alcoholic hepatitis.

### Trial design {8}

This is multicentre, parallel, double-blind, randomised (1:1), placebo-controlled trial.

## Methods: Participants, interventions and outcomes

### Study setting {9}

The study will recruit patients admitted to hospitals with AH at both academic and district general centres in the UK. A list of study sites has been included in the Appendix.

### Eligibility criteria {10}

#### 4.4.1 Inclusion criteria

- Male and female patients aged 18 years or older at screening
- Clinical diagnosis of alcoholic hepatitis at screening:
- Serum bilirubin > 80 μmol/L
- History of excess alcohol (> 80 g/day male, > 60 g/day female) to within 6 weeks before the screening visit
- Less than 4 weeks since admission to hospital at baseline visit
- Maddrey’s Discriminant Function ≥ 32 and MELD ≤ 27 at baseline visit

##### Informed consent

- Women of child-bearing potential have to use an effective contraception method (as specified in the “Contraception and pregnancy” section).

#### 4.4.2 Exclusion criteria

- Alcohol abstinence of > 6 weeks prior to randomisation/baseline visit
- Duration of clinically apparent jaundice > 3 months before the baseline visit
- Other causes of liver disease including:
- Evidence of chronic viral hepatitis (hepatitis B or C)
- Biliary obstruction
- Hepatocellular carcinoma
- Evidence of current malignancy (except non-melanotic skin cancer)
- Previous entry into the study or use of either prednisolone or any systemic steroids (equivalent to a dose of systemic prednisolone > 20 mg) within 6 weeks of screening.
- AST > 500 U/L or ALT > 300 U/L (not compatible with alcoholic hepatitis)
- Patients with a serum creatinine > 220 μmol/L (2.5 mg/dL) or requiring renal support (see below)
- Patients dependent upon inotropic support (adrenaline or noradrenaline). Terlipressin is allowed
- Variceal haemorrhage on this admission
- Untreated sepsis (see below)
- Patients with known hypersensitivity or contraindications to canakinumab
- Patients with cerebral haemorrhage, extensive retinal haemorrhage, acute myocardial infarction (within the last 6 weeks) or severe cardiac arrhythmias (not including atrial fibrillation)
- Pregnant or lactating women
- Patients treated with other IL-1 inhibitors and biologics or any other immunosuppressants within 3 months of study participation.
- Known infection with HIV at screening or randomisation
- History or evidence of tuberculosis (TB) (active or latent) infection
- Active ongoing inflammatory diseases other than AH that might confound the evaluation of the benefit of canakinumab therapy
- Underlying metabolic, haematologic, renal, pulmonary, neurologic, endocrine, cardiac, infectious or gastrointestinal conditions, including neutropaenia (ANC < 1.5) and leukopaenia, which in the opinion of the investigator immune compromises the subject and/or places the subject at unacceptable risk for participation in an immunomodulatory therapy.
- Significant medical problems or diseases, including but not limited to the following: uncontrolled hypertension (≥160/95 mmHg), congestive heart failure [New York Heart Association status of class III or IV], uncontrolled diabetes
- Vaccination with a live vaccine within 3 months before baseline

All baseline assessments and eligibility criteria are implemented before randomisation.

### Who will take informed consent? {26a}

Patients will be approached by the patient’s usual clinical care team. Following referral and identification, the study will be explained to potential participants by the local Principal Investigator (PI) and his/her team and there will be an opportunity for the participant to ask questions. They will be given a Patient Information Sheet (PIS) and will be given 24 h to consider the study (or less if the patient feels that they do not need this long to decide whether to participate), prior to giving their informed consent. Patients will be given a copy of the signed Informed Consent Form (ICF).

Potential patients for the trial who present with hepatic encephalopathy may be unable to consent for themselves but are not excluded from the trial. Such patients should be considered for the trial as a patient group likely to receive maximum benefit from the trial interventions. Special arrangements are in place to ensure that the interests of such patients are protected. For patients unable to consent for themselves, the decision on whether to consent to participation in a trial will be taken by a legal representative. The legal representative is independent of the research team and will act based on the person’s presumed wishes. The legal representative may be personal or professional. A personal legal representative is not connected with the conduct of the trial but is suitable to act as the legal representative by virtue of their relationship with the adult and is available and willing to do so. The professional legal representative is also not connected with the conduct of the trial but who is the doctor primarily responsible for the adult’s medical treatment, or a person nominated by the relevant health care provider (e.g. an acute NHS Trust or Health Board). A professional legal representative may be approached if no suitable personal legal representative is available. The appropriate legal representative will be provided with the approved Legal Representative Information Sheet and Legal Representative Informed Consent Form, to document the consent process. The consent given by the legal representative remains valid in law until such time as the patient recovers capacity. At this point, the patient will be informed about the trial and asked to decide whether they want to continue in the trial, and consent to continue will be sought from the patient themselves.

Professional legal representation will be from experienced clinicians who are not involved in the ISAIAH study. Guidance for this is published by the Department of Health, Guidance on nominating a consultee for research involving adults who lack capacity to consent (Department of Health, Feb 2008. Retrieved from: https://webarchive.nationalarchives.gov.uk/20130123193236/ and http://www.dh.gov.uk/en/Publicationsandstatistics/Publications/PublicationsPolicyAndGuidance/DH_083131).

### Additional consent provisions for collection and use of participant data and biological specimens {26b}

Informed consent forms will include the option to consent for the collection, and use of, participant data and biological specimens in ancillary studies. These include analyses of hepatic gene expression profiles and serum biomarkers of disease.

## Interventions

### Explanation for the choice of comparators {6b}

The comparator is placebo infusions of 100 mL 5% dextrose. The composition of the placebo infusion has been chosen to match the vehicle solution for canakinumab-treated patients but without active drugs.

### Intervention description {11a}

A single dose of 3 mg/kg canakinumab or an identical placebo will be administered intravenously at baseline (day 1). Canakinumab will be made up by dilution in 100 ml 5% dextrose by unblinded research personnel at each site. The drug or placebo (5% dextrose alone) will be labelled by the designated unblinded team member.

Patients with AST > 2 × ULN on day 28 will receive a second dose administered i.v. on day 28:

- Patients randomised to placebo at baseline with receive placebo at day 28 if AST>× 2 ULN at Day 28.
- Patients randomised to canakinumab at baseline will receive canakinumab at day 28 if AST >× 2 ULN at day 28.

### Criteria for discontinuing or modifying allocated interventions {11b}

The second dose of the study drug (or placebo) should not be administered if the patient experienced an incident of infection during the prior 28 days, MELD > 27 or in cases where the PI is concerned about the patient’s condition deteriorating. Study treatment for pregnant participants must be discontinued immediately.

Participants that fall into one or more of the following categories, which are indications for treatment discontinuation, are not considered as withdrawn or lost from the study:

- Patients who ask to stop study treatment
- Pregnancy
- Psychosis or persisting psychotic symptoms for more than 7 days, that cannot be explained by alcohol withdrawal
- Any event which in the judgement of the PI makes further study treatment inadvisable
- Serious adverse event (SAE) requiring discontinuation of treatment.

They should continue to attend all follow-up study assessments as per protocol, unless they are subsequently withdrawn, lost to follow up or die.

### Strategies to improve adherence to interventions {11c}

The first dose of the investigational medicinal product (IMP) will be a single infusion given whilst the patient is hospitalised for AH. Administration of the second dose of IMP, if required, relies on attendance at the day 28 visit, which in most cases will be an outpatient visit. Participants will be contacted and reminded by telephone prior to day 28 to minimise the number of patients who should receive a second dose of IMP but do not attend the day 28 visit.

### Relevant concomitant care permitted or prohibited during the trial {11d}

Concomitant medication may be given as medically indicated, including alcohol withdrawal and nutritional therapy. Patients who have an infection at screening or at any time during the study should be treated with antimicrobials. Antimicrobials should be continued or given for the first 2 weeks after initiation of IMP in all patients.

Previous studies of monoclonal anti-cytokine antibody therapy in AH have resulted in high rates of life-threatening infections when used both alone [18] and in combination with prednisolone therapy [19]. Alternative therapies for AH, such as prednisolone, pentoxifylline or N-acetyl cysteine (NAC) should therefore not be used for the treatment period of the study and other immunosuppressive treatments must also not be used.

### Provisions for post-trial care {30}

#### Harm and complaints

Imperial College London is the study sponsor and has civil liability insurance to cover the study in all participating centres. Imperial College London also holds negligent harm and non-negligent harm insurance policies which apply to this study.

Participants who wish to complain or have any concerns about any aspect of their treatment during this study should immediately inform the local PI, the local Patient Advisory Liaison Service or the Imperial Academic Health Science Centre Joint Research Office.

The PI will notify the Imperial Clinical Trials Unit (ICTU) of any death or adverse event occurring at any time after a subject has discontinued or terminated study participation that may reasonably be related to this study.

#### Contraception and pregnancy

Reliable contraception should be maintained throughout the study. The following methods of contraception are accepted:

(i) Total abstinence (when this is in line with the preferred and usual lifestyle of the subject). Periodic abstinence (e.g. calendar, ovulation, symptothermal, post-ovulation methods) and withdrawal are not acceptable methods of contraception.
(ii) Female sterilisation (have had surgical bilateral oophorectomy with or without hysterectomy) or tubal ligation at least 6 weeks before taking study treatment. In the case of oophorectomy alone, only when the reproductive status of the woman has been confirmed by follow up hormone level assessment.
(iii) Male sterilisation (at least 6 months prior to screening). For female subjects on the study, the vasectomized male partner should be the sole partner for that subject.
(iv) Barrier methods of contraception: Condom or occlusive cap (diaphragm or cervical/vault caps) with spermicidal foam/gel/film/cream/vaginal suppository.
(v) Use of oral, injected or implanted hormonal methods of contraception or placement of an intrauterine device (IUD) or intrauterine system (IUS) or other forms of hormonal contraception that have comparable efficacy (failure rate < 1%), for example, hormone vaginal ring or transdermal hormone contraception. In the case of the use of oral contraception, women should have been stable on the same pill for a minimum of 3 months before taking study treatment.

Pregnancy is tested with serum bhCG measurement at screening; pregnant patients are excluded from participation. If a participant falls pregnant on the study, then no further doses of IMP should be administered. If a subject or his partner becomes pregnant whilst taking part in the trial or during a stage where the foetus could have been exposed to an IMP, the Investigator must ensure that the subject and the subject’s healthcare professional are aware that follow-up information is required on the outcome of the pregnancy. If the subject leaves the area, their new healthcare professional should also be informed. Each pregnancy occurring whilst the patient is on study treatment must be reported to ICTU within 24 h of learning of its occurrence. Any SAE experienced during the pregnancy and unrelated to the pregnancy must be reported on a SAE form. The pregnancy should be followed up to determine the outcome, including spontaneous or voluntary termination, details of the birth, and the presence or absence of any birth defects, congenital abnormalities, or maternal and/or newborn complications. Pregnancy follow-up should be recorded on the same form and should include an assessment of the possible relationship to the investigational treatment.

### Outcomes {12}

#### Primary end point

Histological improvement of AH on liver biopsy after 28 days of treatment compared to baseline. Histological improvement is defined as any reduction in lobular inflammation (regardless of cell type) and will be a binary outcome measure (yes or no). This end-point has been chosen because it is unlikely to be affected by concurrent disease complications, such as infection and acute kidney injury, that might otherwise confound clinical outcome measures. The outcome variable incorporates both histological results at baseline and day 28 as a single adjudication of histological improvement at day 28 from baseline. Three independent histopathologists, masked to the patient allocation of active drug or placebo for each trial participant, will judge whether the Day 28 histology is better, rather than the same or worse, than baseline histology. In the event of discordant histopathologist opinions, RDG will have the casting vote. Patients who suffer deterioration or death before 28 days as a result of alcoholic hepatitis and are therefore unable to undergo a second liver biopsy will be categorised as having no histological improvement.

#### Secondary end points

- Improvement of individual components (polymorphonuclear cell infiltrate, ballooned hepatocytes and steatosis) of alcoholic hepatitis on liver histology from baseline to day 28.
- Changes in the components of Alcoholic Hepatitis Histological Score (AHHS) [20] from baseline to day 28
- Changes in the components of Nonalcoholic fatty liver disease activity score (NAS) score from baseline to day 28
- Changes in hepatic venous pressure gradient (HVPG) between baseline and day 28
- Changes in serum CK18-M30/M65 from baseline to days 7, 14, 21, 28, 42 and 90
- Change in serum bilirubin from baseline to days 7, 14, 28, 21, 42 and 90
- Change in MELD score from baseline to days 7, 14, 21, 28, 42 and 90
- Change in Glasgow alcoholic hepatitis score (GAHS) from baseline to days 7, 14, 21, 28, 42 and 90
- Change in the mDF score from baseline to days 7, 14, 21, 28, 42 and 90
- Lille score at day 7
- Resolution of systemic inflammatory response syndrome (SIRS) at days 7, 14, 21, 28, 42 and 90 in patients with SIRS at baseline
- Incidence of SIRS at days 7, 14, 21, 28, 42 and 90 in patients without SIRS at baseline
- Mortality rate at day 90
- Incidence of infection and sepsis over 90 days
- Incidence of acute kidney injury over 90 days
- Incidence of variceal haemorrhage, ascites or encephalopathy over 90 days
- safety and tolerability of canakinumab
- Serum and plasma biomarkers of hepatic function and inflammation including cytokine profiles which may indicate the degree of response to IL-1b inhibition.
- Changes in CRP over time
- Length of hospital stay

#### Exploratory end points

- Changes in monocyte oxidative burst function over time
- Changes in circulating monocyte phenotype over time
- Changes in circulating bacterial DNA over time
- Changes in transient elastography (Fibroscan) scores from baseline at day 28 and day 90
- Canakinumab PK/PD profile
- Changes in tissue gene expression from baseline to day 28
- Immunohistochemistry
- Gene variant interaction of PNPLA3 with treatment outcome

### Participant timeline {13}

**Table:** 

	Screening	Baseline^**6, 9, 11**^	Day 7^**4**^	Day 14^**4**^	Day 21^**4**^	Day 28^**2, 11**^	Day 42 ^**1**^,^**4**^	Discharge^**10**^	Day 90^**3**^	Unscheduled
Informed consent	X									
Demographic data	X									
Inclusion/exclusion criteria	X	X								
HIV test^	X									
Pregnancy test^+^	X^5^								X	
*Hepatitis B and C serology*^	X									
Genetic sequencing (PNPLA3)	X									
Urinary ethyl glucuronide	X	X				X	X		X	
*Alcohol consumption*	X					X	X		X	
Duration since admission to hospital		X						X		
*Medical history*	X									
Prior/concomitant medications	X	X	X	X	X	X	X	X	X	
*Vital signs*	X	X	X	X	X	X	X	X	X	X
*Physical exam*	X	X	X	X	X	X	X	X	X	
*Weight*	X					X^1^		X	X	
*Height*	X									
*Calorie and protein intake*		X	X	X	X	X	X	X	X	
Randomisation		X								
IMP/placebo administration		X				X				
*Prothrombin time*	X	X	X	X	X	X	X	X	X	
*Haematology*^*%*^	X	X	X	X	X	X	X	X	X	X
*Clinical chemistry*^*%*^	X	X	X	X	X	X	X	X	X	X
Serum CK18-M30/M65 sample		X	X	X	X	X	X	X	X	
*DF, MELD, GAHS scores*	X	X	X	X	X	X	X	X	X	
*Lille score*			X							
*Overt GI haemorrhage assessment*^$^	X	X	X	X	X	X	X	X	X	
*Acute kidney injury assessment*^$^	X	X	X	X	X	X	X	X	X	
EDTA sample for DNA (16S)	X	X	X	X	X	X			X	
PBMC sample~		X	X	X		X			X	
*Infection/sepsis/SIRS screen*	X	X	X	X	X	X	X	X	X	X
*USS Liver assessment*^$^	X									
Fibroscan measurement^$^		X				X			X	
Gene expression sample		X				X				
Liver biopsy (incl. HVPG measurement*)^8^		X				X				
Adverse events		X	X	X	X	X	X	X	X	
PK/PD samples in blood and ascites fluid #		X	X	X	X	X	X	X	X	
Biomarker serum		X	X	X	X	X	X	X	X	
Stool and saliva sample		X		X		X			X	

### Sample size {14}

It is estimated that improvement of histological alcoholic steatohepatitis will occur in 40% of patients treated with placebo and 80% of patients treated with canakinumab. A trial with 80% power to detect a difference at the *P* < 0.05 threshold would require 23 patients in each arm, 46 in total. Assuming a drop-out rate of 10%, 52 patients in total (26 patients per group) will be recruited. Recruitment will be continued until we have paired biopsies in 48 patients which may require a maximum of 56 patients.

Patients may be randomised and treated before histology result is available. If the histology is not consistent with steatohepatitis, the patient will be withdrawn, and another patient recruited. The withdrawn patient will continue follow-up for safety.

### Recruitment {15}

Regular investigator meetings will be held to support trial recruitment and evaluate any recruitment challenges. Additional study centres may be adopted to meet recruitment targets.

## Assignment of interventions: allocation

### Sequence generation {16a}

Eligible patients will be randomised to receive active drug or placebo 1:1 via the inform electronic randomisation system (Oracle, CA, USA). Block randomisation will be used with variable block sizes to assist in concealing allocation. Randomisation is blinded to site staff except for designated unblinded study personnel and the patient, by means of a unique code.

### Concealment mechanism {16b}

Randomly allocated treatments are blinded to site staff and participants, except for designated unblinded study personnel, by means of a unique code which will be displayed by the Inform electronic randomisation system. Allocated participants will also be sequentially numbered unrelated to treatment allocation.

### Implementation {16c}

The allocation sequence (randomisation list) is prepared by the senior statistician and the final randomisation list is independently generated by an independent statistician. Blinded and delegated study staff will enrol participants. Assignment of intervention (active treatment or placebo) to participant will be performed by delegated unblinded site staff.

## Assignment of interventions: blinding

### Who will be blinded {17a}

After assignment to interventions, the trial participants, care providers, outcome assessors and data analysts will be blinded and will not have access to the randomisation list that would permit unblinding.

The trial team at each site will randomise each new participant using the InForm electronic system. This will provide a unique code, the Study Drug ID, to the trial team for that patient (that does not identify the treatment allocation). The Study Drug ID will be used by the site pharmacy to assign active drugs or placebo according to a pre-specified randomisation list. This will be double-checked by a second pharmacist against the code on InForm and the code on the randomisation list to reduce the risk of error. The pharmacist will then either prepare the infusion bag at trial pharmacy or dispense the IMP or placebo to a designated unblinded research member who will then make up the infusion bag and label it with the Study Drug ID. The Study Drug ID will be further verified by another unblinded member of the research team before administration by a blinded nurse or doctor.

### Procedure for unblinding if needed {17b}

Unblinding may be permitted in the event of a medical emergency where breaking the blind is required to provide medical care to the subject.

Local PIs will have access to a mechanism that permits rapid unblinding should they feel this is necessary and are unable to contact the study team. Local SOPs describing the emergency unblinding procedure will be in place. The chief investigator recommends, but does not require, that the investigator contact him before breaking the blind. The rationale for unblinding must be clearly explained in source documentation and on the electronic case report form (eCRF), along with the date on which the treatment assignment was obtained.

## Data collection and management

### Plans for assessment and collection of outcomes {18a}

The principal means of data collection from participant visits will be Electronic Data Capture (EDC) via the internet using the InForm database. Data is entered into the EDC system by site personnel. All source data recorded in the electronic case report form (eCRF) will be signed by the Investigator or his/her appropriate designee. All changes made following the electronic signing will have an electronic audit trail with a signature and date. Specific instructions and further details will be outlined in the eCRF manual.

Source documents include original documents related to the trial, to medical treatment and to the history of the participant, and adequate source documentation will be maintained to allow reliable verification and validation of the trial data. The requirements for source data for this trial will be outlined in the study Monitoring Plan.

Blood samples for all biochemistry and haematology assessments should be collected in accordance with routine clinical practice. The specific tests for clinical chemistry and haematology are specified in Table 1. Samples will be analysed in the routine clinical laboratories at participating sites and reported via the usual hospital route. All study-related results should always be reviewed, signed and dated by a study clinician.

**Table 1:** Clinical chemistry and haematology panel for the ISAIAH trial

Biochemistry	Haematology
• Albumin • Alkaline phosphatase (ALP) • Total serum bilirubin • Total cholesterol • Low-density lipoprotein cholesterol (LDLC) • High-density lipoprotein cholesterol (HDL-C) • Creatinine • Creatine kinase (CK) • Aspartate aminotransferase (AST) • Alanine aminitransferase (ALT) • α-amylase • Sodium • Potassium • Lactate dehydrogenase (LDH) • Triglycerides • C-reactive protein (CRP) • eGFR • Gamma-GT • Urea • Transferrin	• Heamoglobin • White blood cell count • Lymphocytes • Monocytes • Neutrophils • Platelet count • Prothrombin time • INR • Prothrombin time control

Reported laboratory results should be transcribed to the eCRF within 2 working days of the receipt of hospital report. However, the day 28 assessment must be entered in eCRF on the same day as the AST assessment on day 28 which determines whether the patient will be administered the second dose of IMP that same day. The clinical chemistry and haematology samples are collected at every patient visit. That is, screening and baseline, and days 7, 14, 21, 28, 42, at discharge and day 90.

### Plans to promote participant retention and complete follow-up {18b}

#### Patients lost

The PI will make every reasonable effort to keep each patient on the study. Patients who are withdrawn, lost to follow-up or die will have no further follow-up visits and no further data collected.

Patients who withdraw their consent at any point in the study fall into one of two categories:

- Those who allow their data (collected up to the point of withdrawal) to be used

OR

b) Those who do not allow the use of any of their data collected prior to withdrawal.

Patients who are lost to follow up are those who do not attend a follow-up assessment after site staff have attempted to contact the patient at least twice, e.g. by telephone. These patients have not withdrawn their consent and so the data already collected for them may be used and therefore needs returning in the usual manner.

Patients who had died have not withdrawn their consent and therefore the data already collected for them may be used.

#### Patients not lost

Patients who require treatment discontinuation only are not considered lost from the study. They should continue to attend all follow-up study assessments as per protocol. The indications for treatment discontinuation are:

- Patients who ask to stop study treatment
- Pregnancy
- Psychosis or persisting psychotic symptoms for more than 7 days, that cannot be explained by alcohol withdrawal
- Any event which in the judgement of the PI makes further study treatment inadvisable
- SAE requiring discontinuation of treatment.

Stopping treatment, for whatever reason, does not mean the patients are withdrawn or lost from the study, they should continue to attend all follow-up assessments.

#### Patients with histology not consistent with steatohepatitis at screening liver biopsy

These patients are withdrawn after randomisation. The follow-up period is 28 days. They do not undergo a second biopsy nor further treatment at 28 days. Data will be reported separately in the AE and SAE analysis.

### Data management {19}

All personnel involved in the study will observe or work within the confines of the local data protection guidelines that have been documented in the ISAIAH Data Protection Impact Assessment.

Data collected into eCRFs will be verified by designated Trial Monitors according to a pre-specified Monitoring Plan. Range checks for key data values are built into the eCRF software.

The CI will retain essential documents until notified by the Sponsor, and for at least 10 years after study completion*.* Patient files and other source data (including copies of protocols, CRFs, original reports of test results, IMP dispensing logs, correspondence, records of informed consent, and other documents pertaining to the conduct of the study) will be retained. Documents will be stored in such a way that they can be accessed/data retrieved at a later date.

No study document will be destroyed without a prior written agreement between the Sponsor and the investigator. Should the investigator wish to assign the study records to another party or move them to another location, a written agreement must be obtained from the Sponsor.

### Confidentiality {27}

The PI will ensure that the subject’s confidentiality is maintained. On the CRF or other documents submitted to the Sponsors, subjects will be identified by a subject ID number only. Documents that are not submitted to the Sponsor (e.g. signed informed consent form) should be kept in a strictly confidential file by the PI.

The PI shall permit direct access to subjects’ records and source document for the purposes of monitoring, auditing, or inspection by the Sponsor, authorised representatives of the Sponsor, Regulatory Authorities and Research Ethics Committee (REC).

It is the PI’s responsibility to inform the subject’s General Practitioner by letter that the subject is taking part in the study provided the subject agrees to this, and information to this effect is included in the Patient Information Sheet and Informed Consent. A copy of the letter should be filed in the research record for the patient at sites.

### Plans for collection, laboratory evaluation and storage of biological specimens for genetic or molecular analysis in this trial/future use {33}

Biological specimens for exploratory analyses will be collected concurrently with biological specimens for evaluation of secondary endpoints at individual visits. Peripheral blood mononuclear cells and serum will be harvested from fresh samples and stored in liquid nitrogen and/or − 80° for subsequent analyses.

The exploratory endpoints are listed below:

- Changes in monocyte oxidative burst function over time
- Changes in circulating monocyte phenotype over time
- Changes in circulating bacterial DNA over time
- Changes in transient elastography (Fibroscan) scores from baseline at day 28 and day 90
- Canakinumab PK/PD profile
- Changes in tissue gene expression from baseline to Day 28
- Immunohistochemistry
- Gene variant interaction of PNPLA3 with treatment outcome

## Statistical methods

### Statistical methods for primary and secondary outcomes {20a}

We have identified three populations of interest:

A. The *intention-to-treat (ITT) population:* all patients who have positive histology at screening regardless of whether treatment is completed;
B. A *per-protocol population*: patient with positive histology at screening *and* who completed at least the first dose of IMP;
C. The population with *negative histology at screening*: these patients are withdrawn from the study after randomisation and will be followed up for safety only and not efficacy. *This population of patients do not have AH and would have been excluded if the results of the biopsy were known before randomisation.*

All summaries and analyses will be on the ITT population (population A) unless otherwise specified.

The observed 28-day histological improvement, by treatment group, will be presented for all patients in the ITT population (population A). The primary analysis will test the null hypothesis using a chi-square test. The difference in proportions of histological improvement between treatment groups with 95% confidence intervals will be provided. The primary end point analysis will be repeated for the per-protocol population (population B).

Secondary end point analyses are outlined below in Table 2; further detail is available in the SAP (on request). All statistical tests will be two-tailed with 5% significance level. Continuous secondary outcomes which refer to change in scores are analysed using ANCOVA models, using day 28 or day 90 measurement as the outcome variable, and are adjusted for baseline values in each model.

**Table 2:**
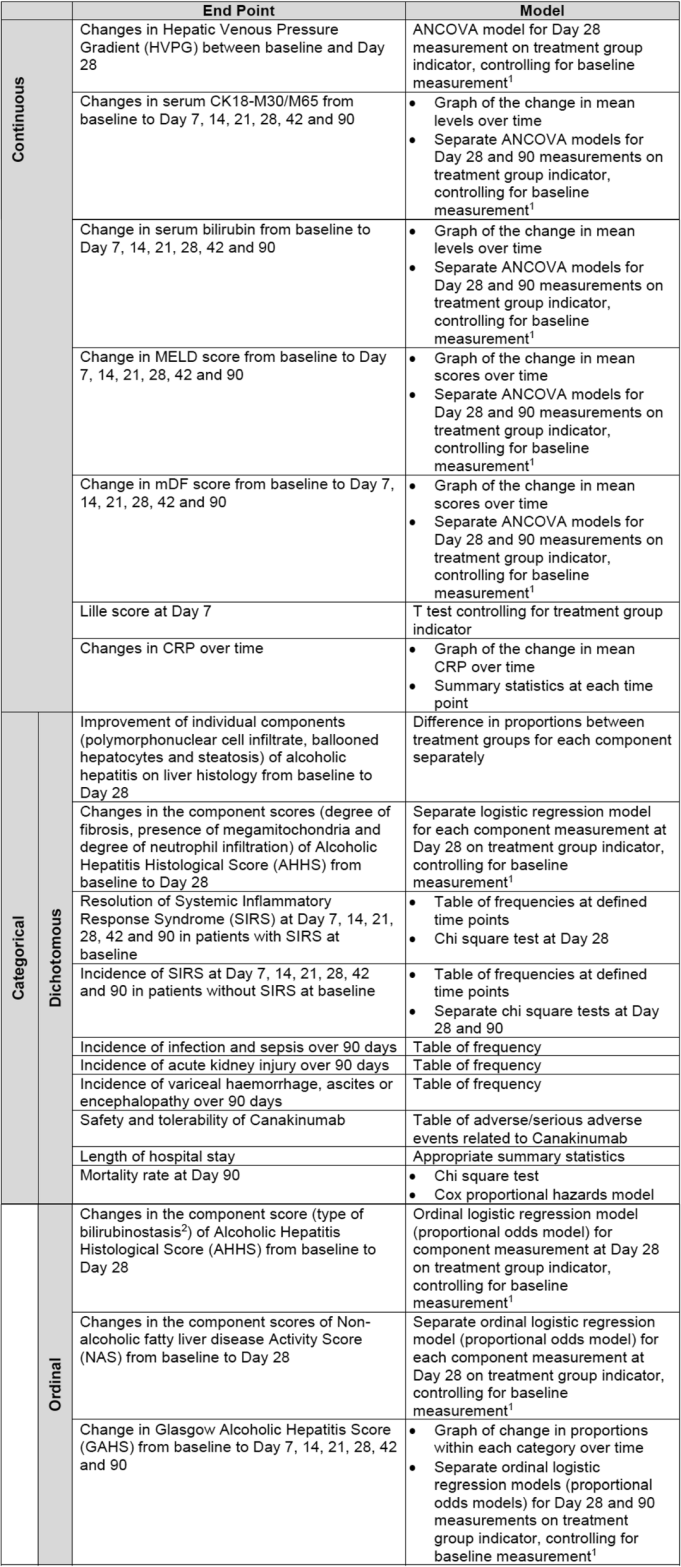
Summary of secondary end point analyses

^1^Baselinemeasurement refers to the baseline measurement of the variable being analysed

^2^Other component scores of AHHS are included in the analysis of dichotomous variables

### Interim analyses {21b}

No formal interim analysis is planned for this trial.

### Methods for additional analyses (e.g. subgroup analyses) {20b}

As a secondary analysis of the primary end point, several univariate logistic regression analyses will be performed for 28-day histological improvement, adjusting for pre-defined baseline variables of clinical disease severity (MELD score); histological disease severity (AHHS score); age; and gender. A separate model will be fitted for each, and odds ratios and 95% CIs produced from each model will be presented. Next, a multivariable logistic regression analysis will be performed for 28-day histological improvement that will include variables that were statistically significant in the univariate analysis plus a variable for the treatment group indicator. If any prognostic scores are significant in the univariate analysis, then the individual components of the scores shall be fitted to the multivariable model rather than the composite scores.

### Methods in analysis to handle protocol non-adherence and any statistical methods to handle missing data {20c}

Please see the “Statistical methods for primary and secondary outcomes {20a}” section for definitions of populations of interest and how they are separately analysed.

The reasons for missing primary end point data and the relationship to study treatment will be presented. It is expected that, at most, 6 participants will have missing primary outcome data, due to an estimated rate of dropout of 10%. Missing primary outcome data will be assumed to be missing completely at random. To test whether this assumption is robust, a sensitivity analysis will be conducted. This analysis will focus on missing data whose missingness is assumed to be related to the study treatment. Constructed scenarios for the missing binary histological improvement outcome data will be considered: worst case; best case and intermediate cases. These scenarios assume that the missing data may not be missing completely at random. Each of these constructed scenarios will be evaluated separately for their potential impact on the mean treatment effect between active drug and placebo.

1. A *worst-case scenario* will explicitly assume that all missing primary outcome data in the control group were successes (histological improvement occurred), and all missing primary outcome data in the treatment group were failures (histological improvement did not occur).
2. A *best-case scenario* will explicitly assume that all missing primary outcome data in the control group were failures (histological improvement did not occur), and all missing primary outcome data in the treatment group were successes (histological improvement occurred).
3. All *intermediate-case scenarios* will explicitly assume all possible allocations of the binary missing primary outcome values in both treatment groups.

The estimated treatment effect and 95% confidence interval for each constructed scenario will be presented, with the statistical significance of each treatment effect estimate summarised by the corresponding *p* value. The point in the constructed scenarios at which the treatment effect changes in statistical significance, knowns as the “tipping point”, will be identified.

### Plans to give access to the full protocol, participant level-data and statistical code {31c}

Verbal or written discussion of results prior to study completion and full reporting should only be undertaken with written consent from the Sponsor. All information obtained as a result of the study will be regarded as confidential, at least until appropriate analysis and review by the investigator(s) are completed.

The results may be published or presented by the investigator(s), but the Sponsor will be given the opportunity to review and comment on any such results before any presentations or publications are produced. All publications and presentations relating to the study will be authorised by the Trial Management Group. Authorship will be determined according to the internationally agreed criteria for authorship (www.icmje.org). Authorship of parallel studies initiated outside of the Trial Management Group will be according to the individuals involved in the project but must acknowledge the contribution of the Trial Management Group and the Study Coordination Centre.

Novartis, as study funder, will be informed of study publications as per the contract between Novartis and Imperial College.

## Oversight and monitoring

### Composition of the coordinating centre and trial steering committee {5d}

The day-to-day management of the trial will be co-ordinated through the Imperial Clinical Trials Unit and the Chief Investigator. A Trial Management Group (TMG) will also be convened including the Chief Investigator, co-investigators and key collaborators, trial statistician and trial manager. The TMG will be responsible for operational issues including recruitment and other practical aspects of the trial.

A Trial Steering Committee (TSC) will be convened including as a minimum an independent Chair, 2 independent clinicians, a lay representative (also independent), the Chief Investigator and Trial Manager. The role of the TSC is to provide overall supervision of trial conduct and progress. A TSC Charter will be devised to list the roles and responsibilities of the TSC members. Frequency of meetings will be defined in the Charter. The first TSC meeting to take place following the start of recruitment will be after the first 10 patients have been randomised to the study and thereafter frequency of meetings will be determined by the DMEC and TSC based on this initial data.

### Composition of the data monitoring committee, its role and reporting structure {21a}

An independent Data Monitoring and Ethical Committee (DMEC) will be set up to monitor progress, patient safety and any ethical issues involved in this trial. They will review trial progress, recruitment rates, event rates and safety data. A separate charter will be drawn up defining their exact remit and criteria for reporting to the trial steering committee. Frequency of meetings will be defined in the Charter. The first DMEC meeting to take place following the start of recruitment will be after the first 10 patients have been randomised to the study and thereafter frequency of meetings will be determined by the DMEC and TSC based on this initial data.

### Adverse event reporting and harms {22}

The Imperial Clinical Trials Unit (ICTU) has been delegated by the Sponsor to undertake all sponsor duties relating to pharmacovigilance. All non-serious AR/AEs, whether expected or not, should be recorded in the adverse event section of the relevant case report form. All new SAEs regardless of causality, occurring after the patient has signed informed consent and until the last patient visit must be reported to ICTU within 24 h of learning of its occurrence and must also be recorded on adverse event case report form (AE CRF) within the InForm database. Recurrent episodes, complications, or progression of the initial SAE must be reported as a follow-up to the original episode, regardless of when the event occurs. This report must be submitted according to the study-specific reporting procedures. An SAE that is considered completely unrelated to a previously reported one should be reported separately as a new event.

At each contact with the subject during the treatment period, the Investigator must seek information on adverse events by specific questioning and, as appropriate, by examination. Information elicited should be recorded immediately in the source document, and the AE CRF. All clearly related signs, symptoms, and abnormal diagnostic procedures results should be recorded in the source document using the event terms and grading given in the relevant eCRF pages. The clinical course of each event should be followed until resolution or stabilisation.

Many clinical events are likely to occur which would ordinarily need recording as adverse events. However, events that are recognised and expected complications of the condition (listed in Appendix 1 of study protocol) are exempt from the normal recording procedures, unless they become ‘serious’ by definition.

A pre-existing condition should not be reported as an AE unless the condition worsens during the trial. The condition, however, must be reported in the Medical History Form.

All serious adverse events and reactions must be reported immediately by the Principal Investigator or delegate to ICTU. In turn, ICTU will inform the Sponsor (within 24 h of becoming aware of the event) and the Funder (as soon as becoming aware and not more than 15 calendar days). Summary of product characteristics (SmPC) should be used as the Reference Safety Information. Serious adverse events expected to occur with Canakinumab should be recorded on an SAE/SUSAR Report Form on InForm and the ICTU/Sponsor informed within 24 h. A submitted SAE form on InForm will automatically send alert emails to the Chief Investigator, the Project Manager, and the Sponsor. Adverse Events considered to be expected for Reporting purposes are detailed in Appendix 2 of the study protocol. These reports should be followed by further detailed SAE/SUSAR Report Forms until resolution of the event. The SAE/SUSAR Report Form should be completed as though the patients were taking active form of IMP, even though all parties are blinded.

ICTU will report SUSARs to the regulatory authorities (MHRA and the relevant ethics committees) as follows:

- SUSARs which are fatal or life-threatening will be reported within 7 calendar days of the CTU first becoming aware of the reaction. Any additional relevant information must be reported within a further 8 days (i.e. by day 15).
- SUSARs that are not fatal or life-threatening will be reported within 15 days of the CTU first becoming aware of the reaction.

Annual Safety reports will be submitted to the Sponsor, the Ethics Committee and Regulatory Authority in accordance with regulatory requirements. Novartis Pharmaceuticals UK Limited will be responsible for submission of the Development Safety Update Report (DSUR). The Sponsor (via ICTU) will provide information required by Novartis to include the study in the Novartis DSUR in an integrated manner. Novartis will provide a copy of the submitted DSUR to ICTU for filing in the study TMF.

If any urgent safety measures are taken the CI/Sponsor shall immediately and in any event no later than 3 days from the date the measures are taken, give written notice to the relevant REC of the measures taken and the circumstances giving rise to those measures.

#### Infections

Episodes of infection are of special interest in this study given the mode of action of the intervention and the patient population. Infections will therefore be prospectively recorded in the eCRF. Serious infections will additionally be recorded by SAE reporting. SAE reports will be further subdivided by MedDRA categorisation that will document the site of infection. Infections will be tabulated and presented using both methods of reporting.

### Frequency and plans for auditing trial conduct {23}

Initiation visits will be completed at all trial sites prior to the recruitment of participants and will consist of a review of protocol and trial documents, training with respect to trial procedures (informed consent, SAE reporting, inclusion, and exclusion criteria), review of recruitment strategy, review of site facilities and equipment, essential document receipt, collection and filing, and archiving and inspection.

The study will be monitored periodically by a trial monitor to assess the progress of the study, verify adherence to the protocol, ICH GCP E6 guidelines and other national/international requirements and to review the completeness, accuracy, and consistency of the data. A monitoring plan will be devised based on risk analysis and described in detail in the monitoring manual. A Trial Monitor will visit all sites and facilities where the trial will take place to ensure compliance with the protocol, GCP and local regulatory compliance.

The investigators will allow the monitors to:

- Inspect the site, the facilities, IMP management and materials used for the trial
- Meet all members of the team involved in the trial, and ensure all staff working on the trial are experienced and appropriately trained, and have access to review all of the documents relevant to the trial
- Have access to the electronic case record forms and source data
- Discuss with the investigator and site staff trial progress and any issues on a regular basis

The monitor will ensure that:

- All participant records will be inspected for confirmation of existence, eligibility and informed consent
- There is adherence to the protocol, including consistency with inclusion/exclusion criteria
- There is GCP and regulatory compliance
- Trial Documentation is complete and up to date (e.g. correct versions of documents being used, source data captured) and relevant documents are collected for the Trial Master File (TMF)
- The eCRFs have been completed correctly and accurately, and all entries correspond to data captured in source documents

At the end of the trial, close out visits will be performed by the monitor after the final participant visit has been completed.

Each investigator will also be notified that an audit or inspection may be carried out—by the sponsor, sponsor’s representatives or the host institution, or regulatory authorities—at any time, before, during or after the end of the trial. The investigator must allow the representatives of the audit or inspection team:

- To inspect the site, facilities and material used for the trial
- To meet all members of his/her team involved in the trial
- To have direct access to trial data and source documents
- To consult all of the documents relevant to the trial

If an Investigator is informed of an impending audit or inspection, the trial coordination centre should be notified immediately.

Quality Control will be performed according to Imperial Clinical Trials Unit internal procedures. The study may be audited by a Quality Assurance representative of the Sponsor and/or ICTU. All necessary data and documents will be made available for inspection.

The study may also be subject to inspection and audit by Imperial College London under their remit as Sponsor, the Study Coordination Centre and other regulatory bodies to ensure adherence to GCP.

### Plans for communicating important protocol amendments to relevant parties (e.g. trial participants, ethical committees) {25}

Approved protocol modifications will be communicated to sites by the Trial Manager and relevant site documentation will be updated.

If any urgent safety measures are taken the CI/Sponsor shall immediately and in any event no later than 3 days from the date the measures are taken, give written notice to the relevant REC of the measures taken and the circumstances giving rise to those measures.

If, in the opinion of the Chief Investigator, clinical events indicate that it is not justifiable to continue the trial, the Trial Steering Committee may terminate the trial following consultation with the Sponsor.

### Dissemination plans {31a}

Verbal or written discussion of results prior to study completion and full reporting should only be undertaken with written consent from the Sponsor. All information obtained as a result of the study will be regarded as confidential, at least until appropriate analysis and review by the investigator(s) are completed.

The results may be published or presented by the investigator(s), but the Sponsor will be given the opportunity to review and comment on any such results before any presentations or publications are produced. All publications and presentations relating to the study will be authorised by the Trial Management Group. Authorship will be determined according to the internationally agreed criteria for authorship (www.icmje.org). Authorship of parallel studies initiated outside of the Trial Management Group will be according to the individuals involved in the project but must acknowledge the contribution of the Trial Management Group and the Study Coordination Centre.

Novartis, as study funder, will be informed of study publications as per the contract between Novartis and Imperial College.

Internet and social media will be used to disseminate trial results to wider stakeholders such as healthcare professionals, patients and the public.

## Discussion

The COVID-19 pandemic in 2020 and 2021 presented considerable challenges to the recruitment of participants and their follow up visits and may have increased the proportion of missing data.

## Trial status

The trial was prospectively registered with EudraCT: 2017-003724-79, on 13/4/2018 and found at: https://www.clinicaltrialsregister.eu/ctr-search/search?query=eudract_number:2017-003724-79. It is also registered at ClinicalTrials.gov (NCT03775109).

Recruitment of the first patient first visit was 21^st^ December 2018. Last patient last visit is scheduled for January 2021. The manuscript was first submitted before last patient last visit. Submission of this protocol was not possible earlier because of disruption and staff availability during the COVID-19 pandemic.

Version 5.0 of the protocol was approved 3 September 2020.

## Data Availability

Until appropriate analysis and review by the investigator(s) are completed, all information obtained because of the study will be regarded as confidential. The results may be published or presented by the investigator(s), but the Sponsor will be given the opportunity to review and comment on any such results before any presentations or publications are produced. All publications and presentations relating to the study will further be authorised by the Trial Management Group. Authorship will be determined according to the internationally agreed criteria for authorship (www.icmje.org). Authorship of parallel studies initiated outside of the Trial Management Group will be according to the individuals involved in the project but must acknowledge the contribution of the Trial Management Group and the Study Coordination Centre.

## Abbreviations

AE: Adverse event
AH: Alcoholic hepatitis
AHHS: Alcoholic Hepatitis Histological Score
ALT: Alanine aminotransferase
ANC: Absolute neutrophil count
AR: Adverse reaction
AST: Aspartate aminotransferase
BM: Biomarkers
BP: Blood pressure
CAPS: Cryopyrin-associated periodic syndromes
CI: Chief Investigator
CRF: Case report form
CTU: Clinical Trials Unit
CXR: Chest X-ray
DMEC: Data Monitoring and Ethical Committee
DNA: Deoxyribonucleic acid
DSUR: Development Safety Update Report
eCRF: Electronic case report form
EDC: Electronic Data Capture
FMF: Familial Mediterranean fever
GAHS: Glasgow Alcoholic Hepatitis Score
GCP: Good Clinical Practice
HIDS: Hyperimmunoglobulin D syndrome
HIV: Human immunodeficiency virus
GI: Gastrointestinal
ICMJE: International Committee of Medical Journal Editors
ICTU: Imperial Clinical Trials Unit
IUD: Intrauterine device
IUS: Intrauterine system
IMP: Investigational
LDH: Lactate dehydrogenase
MA: Marketing Authorisation
mDF: Maddrey’s discriminant function
MELD: Model for End-Stage Liver Disease
MHRA: Medicines and Healthcare Products Regulatory Agency
MSU: Mid-stream urine
NAS: Nonalcoholic fatty liver disease activity score
PBMC: Peripheral blood mononuclear cell
PCR: Polymerase chain reaction
PD: Pharmacodynamics
PI: Principal Investigator
PIS: Patient Information Sheet
PK: Pharmacokinetics
QA: Quality Assurance
REC: Research Ethics Committee
SAE: Serious adverse event
SAP: Statistical analysis plan
s.c.: Subcutaneous
SIRS: Systemic inflammatory response syndrome
SJIA: Systemic juvenile idiopathic arthritis
SmPC: Summary of product characteristics
SOP: Standard operating procedure
SSAR: Suspected serious adverse reaction
SUSAR: Suspected unexpected serious adverse reaction
TB: Tuberculosis
TMF: Trial Master File
TMG: Trial Management Group
TRAPS: Tumour necrosis factor receptor associated periodic syndrome
TSC: Trial Steering Committee
ULN: Upper Limit of Normal
UNOS: United Network of Organ Sharing
UTI: Urinary Tract Infection
WHO: World Health Organization
